# Supplementary material for: Testing predictions of inclusive fitness theory in inbreeding relatives with biparental care
Source: Proc Biol Sci. 2019 Dec 4;286(1916):20191933. doi: 10.1098/rspb.2019.1933 (PMC6939262; doi:10.1098/rspb.2019.1933)
Supplement: Methods and Figures [file rspb20191933supp1.pdf]

Electronic Supplementary Materials

Testing predictions of inclusive fitness theory in inbreeding relatives with biparental care

Elizabeth A Gow, Peter Arcese, Danielle Dagenais, Rebecca J Sardell, Scott Wilson, Jane M Reid

Proceedings of the Royal Society B

DOI: 10.1098/rspb.2019.1933

Table of contents:

Appendix S1: Calculation of metrics of parent-offspring relatedness

Figure S1: Schematic illustration of calculation of relatedness

Figure S2: Example calculation of total allelic value (TAV)

Figure S3: Example calculation of lost allelic value (LAV)

Appendix S2: Further details of paternity and pedigree analyses, and observation of feeding rates

Appendix S3: Null model variables, and focal and null variable distributions

Figure S4: Frequency histograms of null and focal variables

Figure S5: Relationships between parental feeding rates and time of season and nestling age

Figure S6: Relationships between parental feeding rates and brood size

Figure S7: Relationships between parental feeding rates and social status

Appendix S4: Relationships between male and female total allelic value (TAV) and brood size

Figure S8: Female and male TAV and brood size

Figure S9: TAV for males versus females within a pair

Appendix S5: Relationships between proportion of extra-pair offspring (P_{EPO}) and other variables

Figure S10. Relationship between P_{EPO} and social status

Figure S11. Relationship between lost allelic value (LAV) and P_{EPO}

Figure S12: Relationships between female and male feeding rates and P_{EPO}

Appendix S6: Supplementary results

Table S1: Null model variables and results from the null model

Table S2: Results from models with TAV, TAV_z and brood size

Table S3: Results from models with focal variables LAV, P_{EPO}, k_{ij}, and f_i and f_j

Appendix S1: Calculation of metrics of parent-offspring relatedness

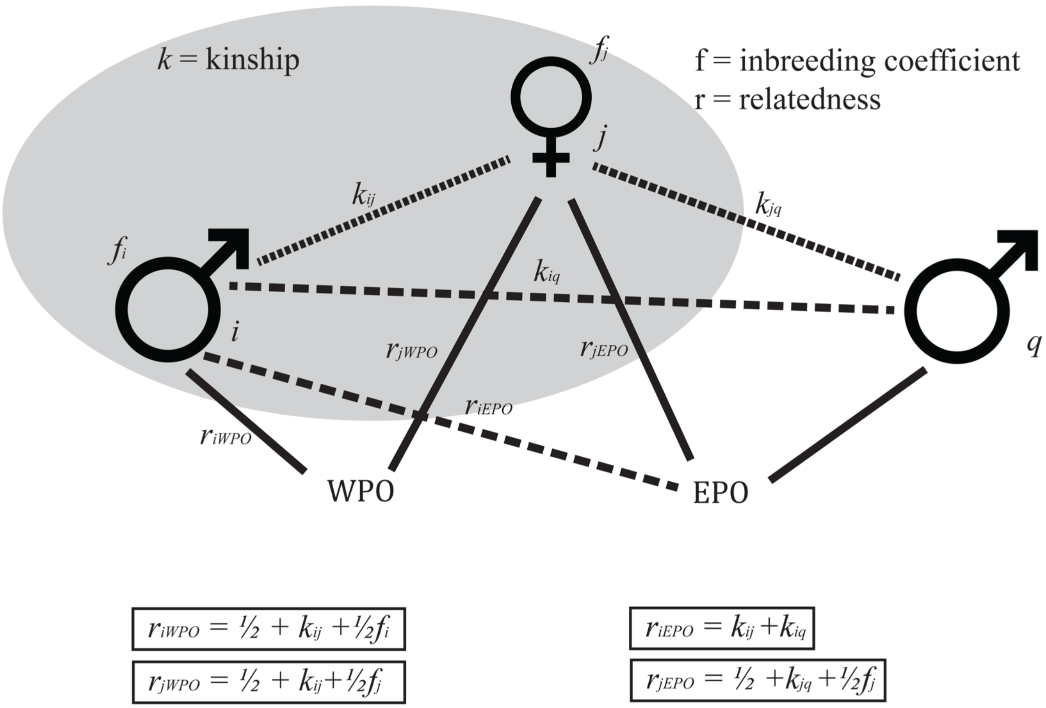

Figure S1. Schematic illustration of calculation of relatedness between a parent and its genetic offspring (i.e., a male's within-pair offspring (WPO) giving r_{iWPO}, or a female's WPO or extra-pair offspring (EPO) giving r_{jWPO} and r_{jEPO}, respectively), and the relatedness between a male and an EPO that it did not sire but could rear (r_{iEPO}). The parameters *i, j,* and *q* denote the identities of the focal male, its socially-paired female, and the female's extra-pair mate (i.e., the sire of the EPO). k_{ij} is the coefficient of kinship between male *i* and his socially-paired female *j,* and is identical to the coefficient of kinship between female *j* and male *i.* k_{iq} is the coefficient of

kinship between *i* and *j*'s extra-pair mate *q*, and k_{jq} is the coefficient of kinship between *j* and *q*. f_i and f_j are *i* and *j*'s own coefficients of inbreeding respectively. The grey oval highlights the social pairing, involving *i* and *j*. Dotted lines indicate mating, solid lines indicate parentage, and dashed lines indicate links between other potentially related individuals.

	WPO	EPO	WPO
♂	$TAV_i = (r_{iWPO} = ½ + k_{ij} + ½ f_i)$	$(r_{iEPO} = k_{ij} + k_{iq})$	$(r_{iWPO} = ½ + k_{ij} + ½ f_i)$
♀	$TAV_j = (r_{jWPO} = ½ + k_{ij} + ½ f_j)$	$(r_{jEPO} = ½ + k_{jq} + ½ f_j)$	$(r_{jWPO} = ½ + k_{ij} + ½ f_j)$

Figure S2. Example calculation of total allelic value (TAV) for a socially-paired male and female rearing a hypothetical brood comprising two within-pair offspring (WPO) and one extra-pair offspring (EPO), and hence total brood size (BS) of three nestlings. TAV is calculated by summing appropriate values of relatedness for each nestling in the brood, calculated with respect to each focal parent using the formulae specified (see also Figure S1). Note that this can generate different values of TAV with respect to a focal male *i* (TAV_i) and his socially-paired female *j* (TAV_j). In general, TAV increases with BS and, conditional on BS, is larger when the parents have higher coefficients of inbreeding (f_j or f_i) and/or higher coefficients of kinship (k_{ij} and/or k_{iq} and/or k_{jq}). All terminology is defined in figure S1.

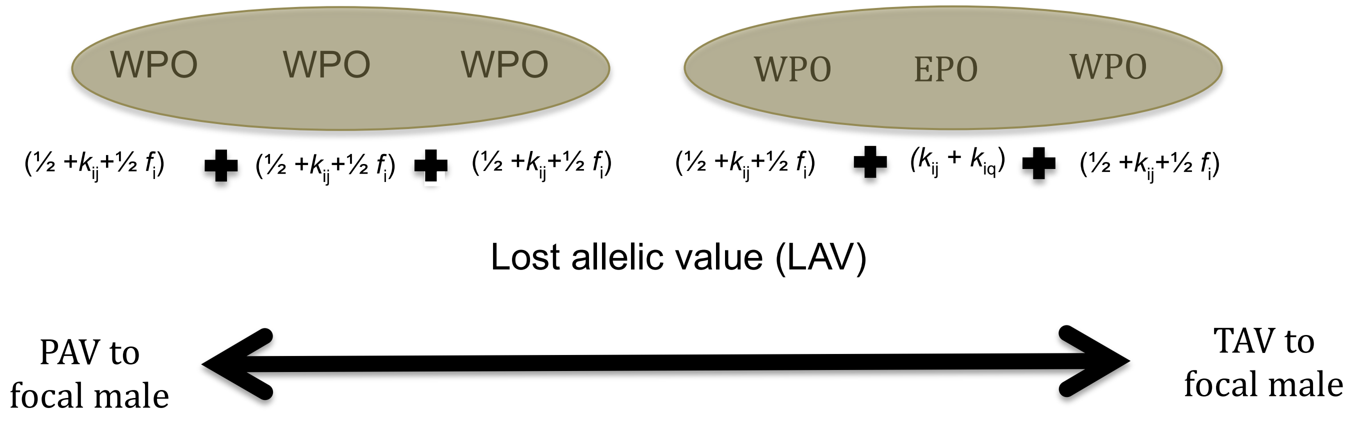

Figure S3. Example calculation of lost allelic value (LAV) as the decrease in total allelic value of a brood to a focal male (TAV_i) resulting from extra-pair paternity (EPP) in offspring produced by the male's socially-paired female. LAV is calculated as PAV-TAV_i, where PAV is the brood's potential allelic value to the focal male, which equals the brood's TAV to the male calculated as if he had sired the entire brood. WPO and EPO denote within-pair and extra-pair offspring respectively. The indices *i, j,* and *q* denote the identities of the focal male, its socially-paired female, and the female's extra-pair mate (i.e., the sire of the EPO). k_{ij} is the coefficient of kinship between male *i* and its socially-paired female *j,* and k_{iq} is the coefficient of kinship between *i* and *j*'s extra-pair mate *q*. LAV can therefore take values between 0 (given zero paternity loss) and ½BS(1+f_i) (given complete paternity loss and k_{iq}=0).

Appendix S2: Further details of paternity and pedigree analyses, and observation of feeding rates

Pedigree construction and analysis

All nestlings and adults ringed since 1993 were genotyped at 160 microsatellite loci [1]. The identities of the genetic parents of each nestling were identified with >99% individual-level statistical confidence [2,3]. Overall, 28% of nestlings were assigned as EPO and 72% as WPO [4]. The social pedigree (i.e., constructed from observed social parentage) was corrected for EPP from 1993 onwards [1,3,5]. Coefficients of kinship (k) between any two individuals, and each individual's own coefficient of inbreeding (f) were calculated from the corrected pedigree using standard algorithms and are relative to a 1975 baseline [2,5,6]. Since the focal individuals have multiple generations of genetically verified ancestors there is very little error in k and f relative to the defined baseline [5]. For reference, values of k of 0, 0.0625, 0.125, 0.25 indicate pairings between unrelated individuals and outbred third-order, second-order, and first-order relatives, respectively. The same values of f refer to offspring of such pairings. Both f and k are expectations, and realized parent-offspring relatedness will deviate from these expectations due to Mendelian sampling variance. However, such variance is reduced by inbreeding, and also reduced across groups of individuals. This implies that deviations between expected and realized relatedness will commonly be relatively small in our system, especially for TAV for broods that contain multiple offspring.

Parental feeding rates

On Mandarte Island, song sparrows construct open cup nests in grass/scrub margins. Individual parents are typically consistent in the route they approach their nest, as determined by the orientation of open vegetation and foraging locations. For each focal nest, typical parental approach routes were determined during extensive observations during incubation (for females)

and early stages of chick feeding (both parents). These observations also served to accustom breeding sparrows to observer presence.

For each feeding rate observation session a single observer approached the territory along established trails, and positioned themselves 15–20m from the focal nest in the best possible position (often on a free-standing ladder or high rock) to retain a clear line of sight of incoming parents while minimizing disturbance. A 10–15 min habituation period was then imposed, during which observers checked for signs of disturbance (e.g. parental alarm calls), and allowed parental behaviour to return to normal after any initial disturbance. If any signs of disturbance were evident, the observer changed position and the habituation period was repeated; however, in most cases no such impacts occurred. The 1-hour session then commenced, and the observer noted the time of arrival of each parent (as identified from their colour-rings). It is unlikely that any visits were missed, due to the close proximity to the nest and consistency in the routes parents took to the nest. However, in three sessions the visiting parent's identity was uncertain during at least one nest visit. These sessions were excluded from analyses. The total of 338 retained sessions spanned the 12-day nestling period (2–5 days, N=112; 6–7 days; N=117; 8–11 days; N=109).

Some nests initiated in the population during the focal periods were excluded from field observations, either because their locations prevented reliable observations without causing disturbance, or because they failed during incubation or early in the chick-rearing period. Observers also attempted to visually score food loads as small, medium or large, but such scoring was not always possible and could not be easily validated. The number of feeding visits per hour was consequently used as the focal metric, and is taken to capture one aspect of parental care. There were no observations of parents visiting nests without food, providing no obvious evidence of 'cheating'. Observations also suggested that parents typically brought relatively equal amounts of food each visit (e.g. either one or two large items or several small items), suggesting that feeding rate likely predicts the amount of food provided to offspring.

Feeding rate data were collected blind to key predictor variables; indeed, the molecular genetic paternity analyses required to calculate TAV, P_{EPO} and LAV were not undertaken until after each breeding season had finished. Extra-pair sires were never observed to visit nests where they had sired extra-pair offspring.

Appendix S3: Null model variables, and focal and null and variable distributions

Over half of the observation sessions were at monogamous nests (N=192), while the rest were split between primary polygynous and secondary polygynous nests (N= 74 and 71, respectively; Fig S4a). Across all 138 observed nests, mean brood size was 2.9±1.0 nestlings (median: 3, range 1-4, Fig. S4b). Mean TAV values were 1.411±0.682 (range 0.063–2.946) for males and 1.785±0.584 (range 0.538–2.998) for females (Fig S4c, d). Mean P_{EPO} was 0.27±0.35 (median 0, range 0–1, 52% zeros; Fig S4e). Mean LAV was 0.208 ± 0.373 (median 0.000, range 0.000–1.913, 50% zeros; Fig S4f). Across all 75 observed female-male pairs, mean k_{ij} was 0.085±0.054 (range 0.000–0.300, Fig S4g). Mean f_i was 0.056 ± 0.038 (range 0.000–0.181) across 55 individual males (Fig S4h) and mean f_j was 0.055 ± 0.035 (range 0.000–0.164) across 65 individual females (Fig S4i).

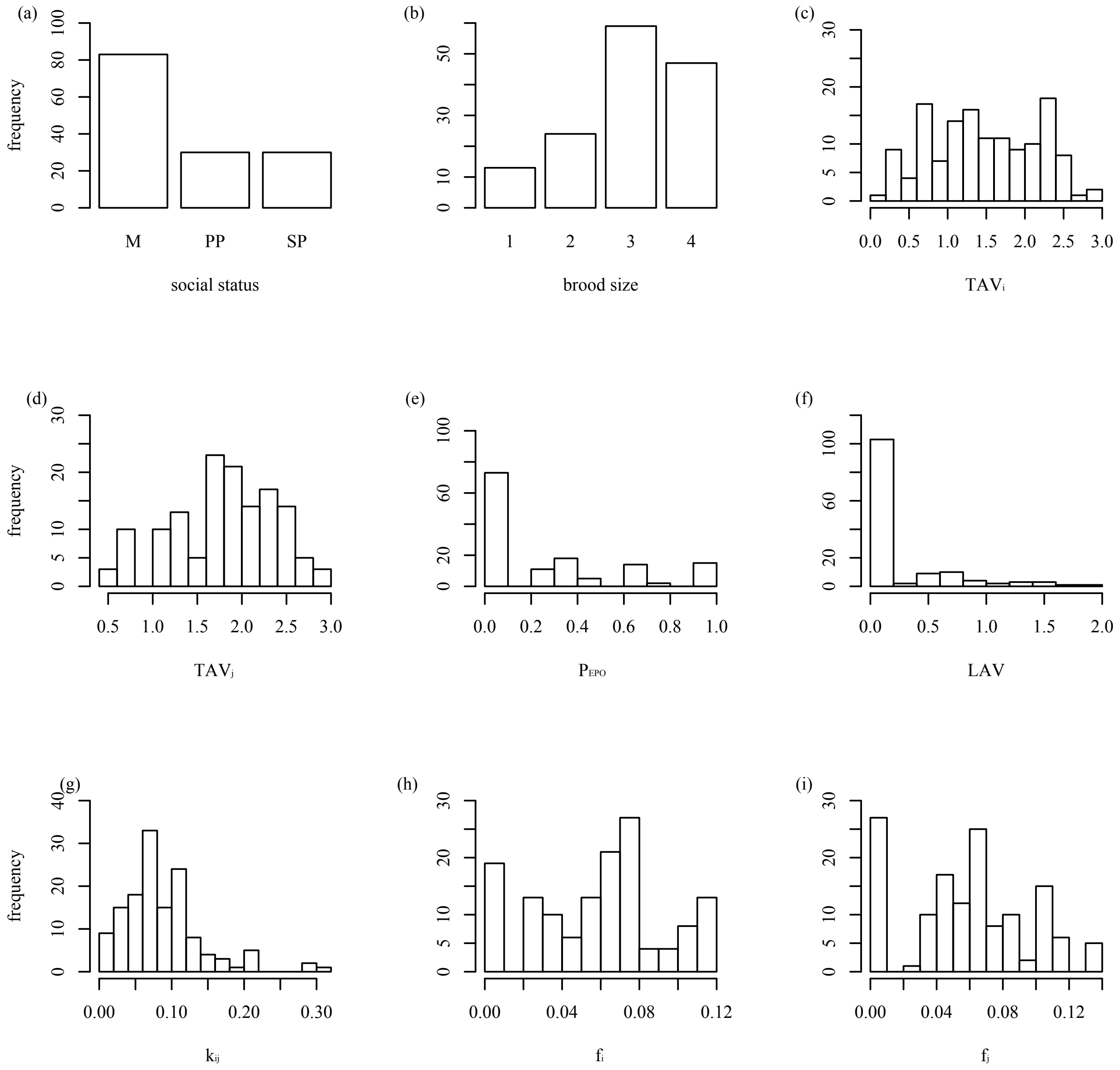
(a)
frequency
M
PP
SP
social status
(b)
brood size
(c)
TAVi
(d)
TAVj
(e)
PEPO
(f)
LAV
(g)
kij
(h)
fi
(i)
fj

142

Figure S4. Frequency histograms (number of individuals) of null and focal variables. Social statuses (a) are defined as monogamous (M), only one female per male; primary polygynous (PP), two females per male territory but the first nest to hatch; secondary polygynous (SP), two or three females per male territory but the second or third nest to hatch. TAV_i (c) and TAV_j (d) are the "total allelic values" for males and females respectively. P_{EPO} is the proportion of extra-pair offspring in a brood (e) and LAV is the "lost allelic value" (f). k_{ij} is the coefficient of kinship between socially-paired mates (g) and male f_i and female f_j (h, i) are the coefficients of inbreeding of the focal male and female.

Null model variables

Both sexes' feeding rates increased with increasing nestling age, but male feeding rates decreased as the breeding season progressed (Fig S5). Models that contained brood size as a linear covariate were slightly better supported than the null model that excluded brood size for males (ΔAIC_c:-0.7) and much more strongly supported for females (ΔAIC_c:-11.1). Models that contained brood size as a continuous variable were better supported than models that contained brood size as a factor for males (ΔAIC_c:+3.6) and females (ΔAIC_c:+7.1; Table S2). Male and female feeding rates increased with brood size (Fig S6). Feeding rates varied with social status, with females feeding more at secondary polygynous than monogamous and primary nests, in contrast males fed less at secondary polygynous nests (Fig S7).

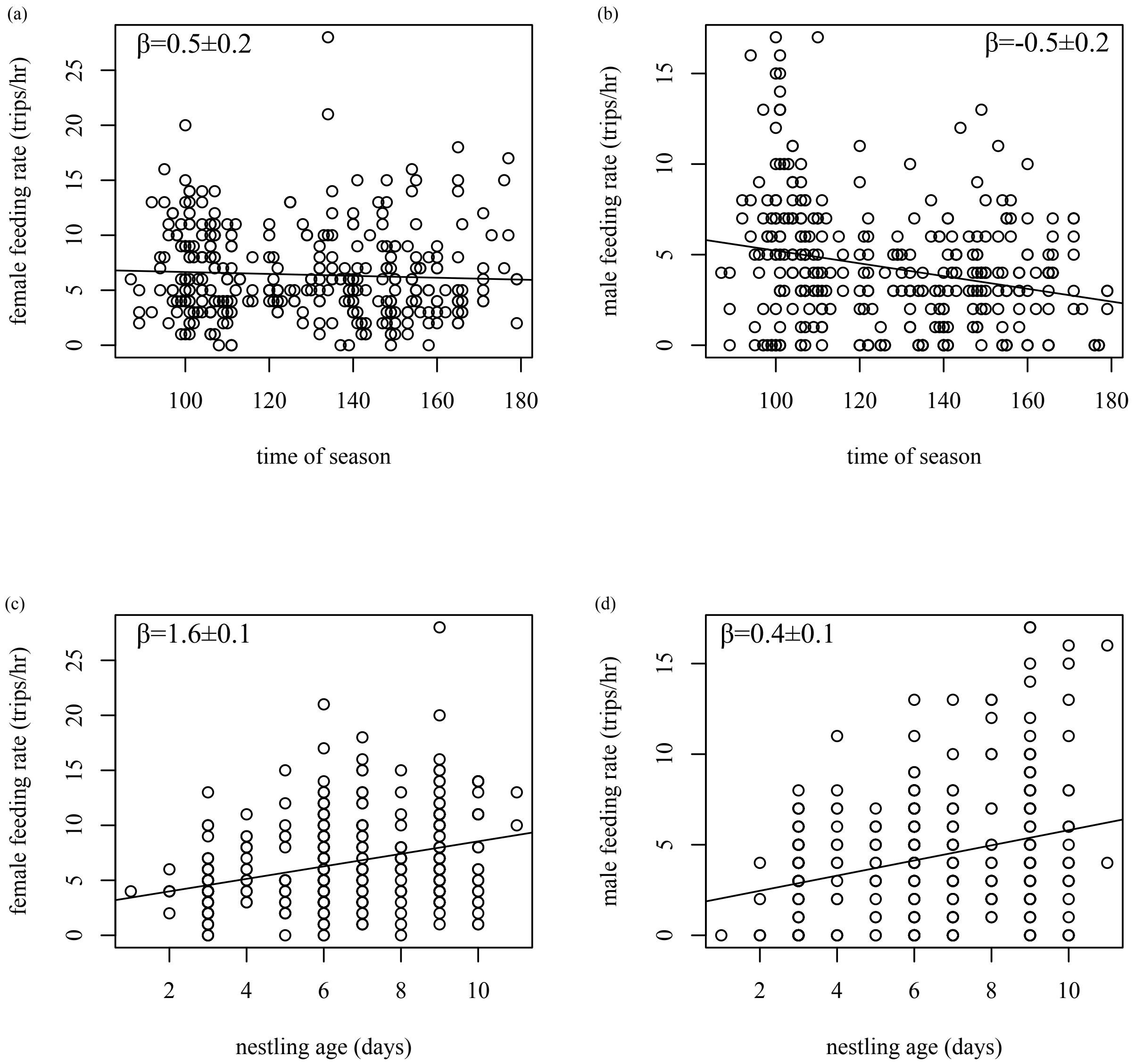

Figure S5. Relationships between male and female feeding rates and time of season (first egg date; a, b) and nestling age (c, d). Points denote individual observation sessions and lines denote linear regressions. Time of season is in Julian dates, with 1 representing January 1. β are the estimates from the null model and BS (as a continuous covariate) ±SE. Statistics are in Appendix S6.

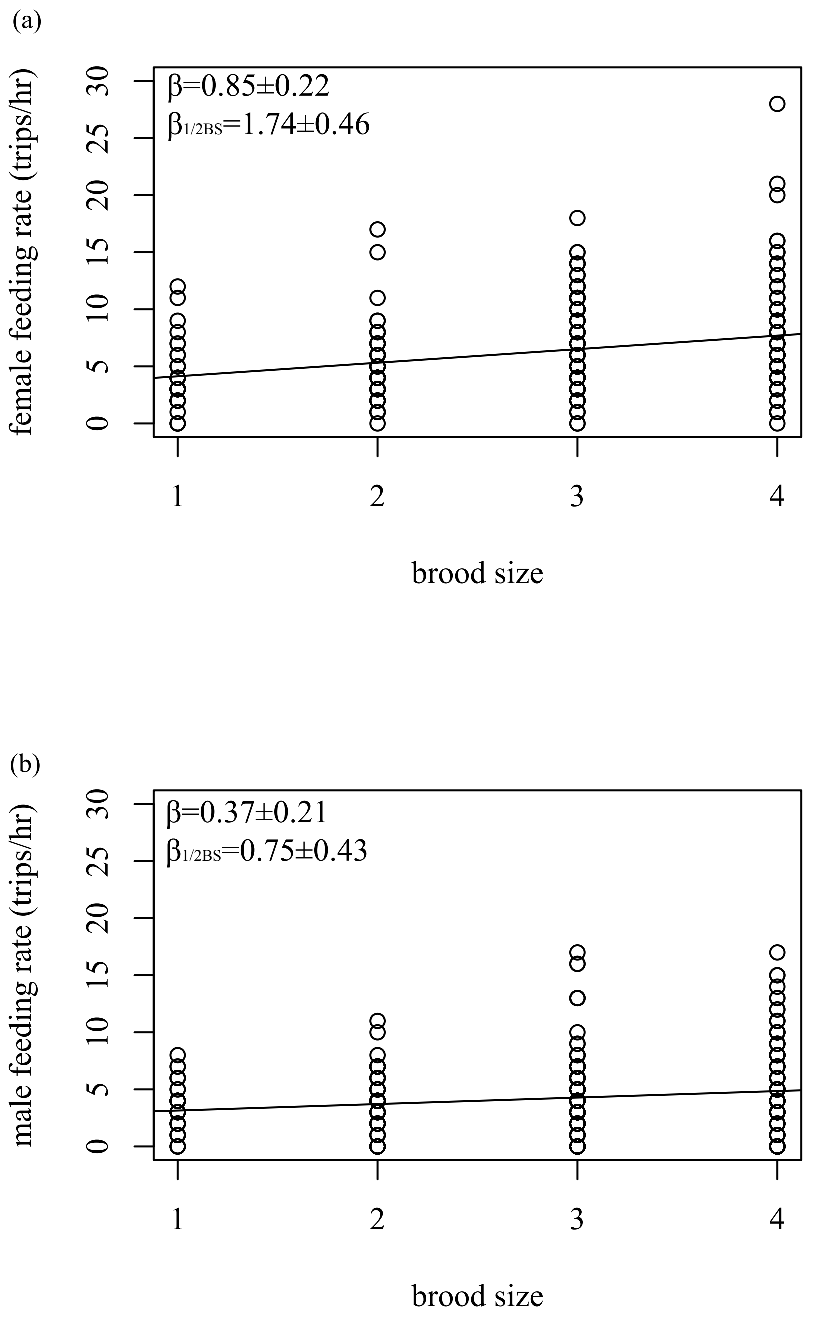

Figure S6. Relationships between female (a) and male (b) feeding rates and brood size. Points denote individual observation sessions and lines denote linear regressions. β are the estimates ±SE from the null model and BS (as a continuous variable). $\beta_{1/2BS}$ indicates the β estimate for a

null model and ½ BS. Slopes of ½ BS were included as they can be compared to those from TAV.

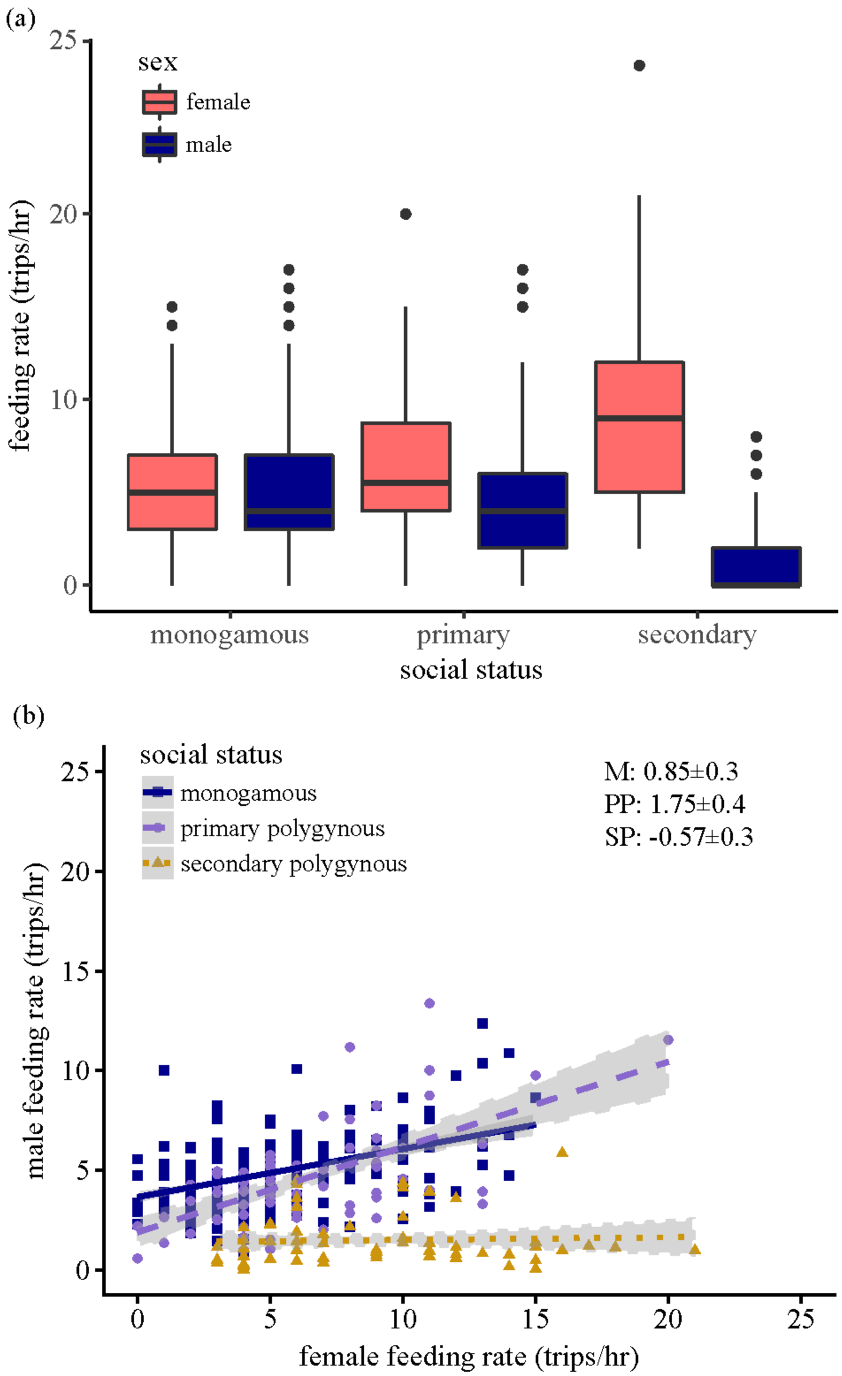

Figure S7. (a) Female and male feeding rates across ‘monogamous’, ‘primary polygynous’ and ‘secondary polygynous’ nests. Boxplots represent the median, upper and lower quartiles, and minimum and maximum values, with outliers depicted by dots. (b) Relationship between male and female feeding rates across nests of different social status. Social statuses are

monogamous (M), primary polygynous (PP), and secondary polygynous (SP). Points represent individual observation sessions, across monogamous (squares, solid line), primary polygynous (circles, dashed line) and secondary polygynous nests (triangles, dotted line). Lines are linear predictions for each social status, and grey-shaded areas are confidence intervals around the line. Numbers on plots represent estimates ± standard errors from the null model and BS (as a continuous variable) for female feeding rates at monogamous, primary and secondary polygynous nests.

Appendix S4: Relationships between male and female total allelic value (TAV) and brood size

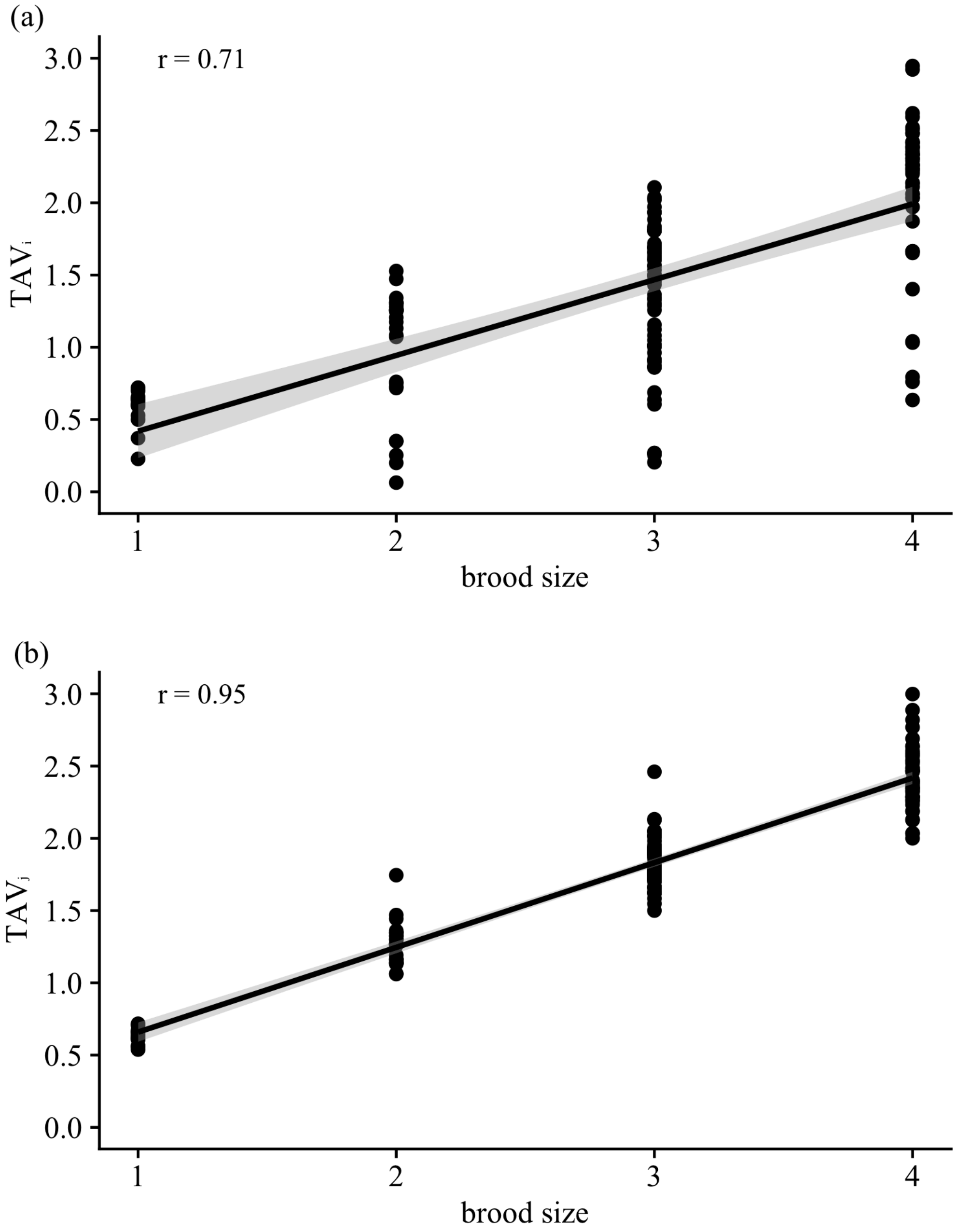

Figure S8. Male (a) and female (b) total allelic values (TAV_i and TAV_j) of a focal brood in relation to brood size. Points denote broods (total N=138), and lines denote linear regressions. r indicates the Pearson correlation coefficient.

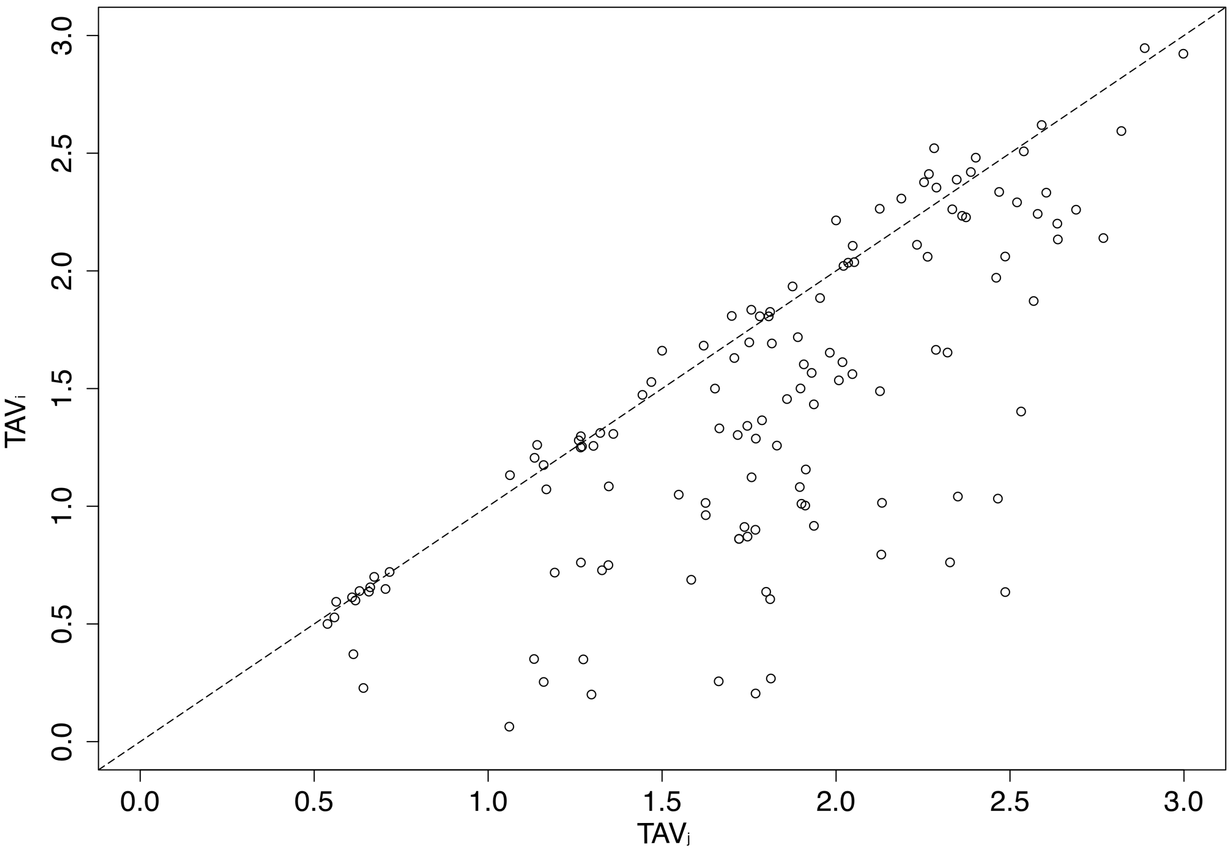

Figure S9. Total allelic values of a focal brood for males (TAV_i) and females (TAV_j) within a pair. Points denote broods (total N=138) and the dashed line indicates equal TAV values for each pair member. See [8] for more detailed consideration of this relationship.

Appendix S5: Relationships between P_{EPO} and other variables

Traditionally, most studies considering socially monogamous systems with extra-pair paternity use the proportion of a brood that comprises extra-pair offspring (P_{EPO}) as a metric to quantify a male's relatedness to a brood. Instead, we define "lost allelic value" (LAV) as a more appropriate metric in systems with reproductive interactions among relatives (Appendix S1). However, to facilitate comparison with existing studies, we also assessed relationships among P_{EPO}, feeding rates, social status, and TAV.

P_{EPO} varied with social status (Fig S10). P_{EPO} was of course strongly positively correlated with LAV (r_p=0.89), but not all values of P_{EPO} and LAV aligned (Fig S11). P_{EPO} was not correlated with TAV_j (i.e., female TAV, r_p=-0.14) but was negatively correlated with TAV_i (i.e., male TAV, r_p=-0.62). Models explaining variation in feeding rates that included P_{EPO} were similarly supported as the null model that also included BS (as a continuous variable) for males (ΔAIC_c=+0.4) but less well supported for females (ΔAIC_c=+1.8); feeding rate decreased with increasing P_{EPO} in males but not females (Fig S12). Models that additionally included P_{EPO} by social status interactions were similarly well supported as the null model for males (ΔAIC_c=+0.4) and less supported for females (ΔAIC_c=+2.3).

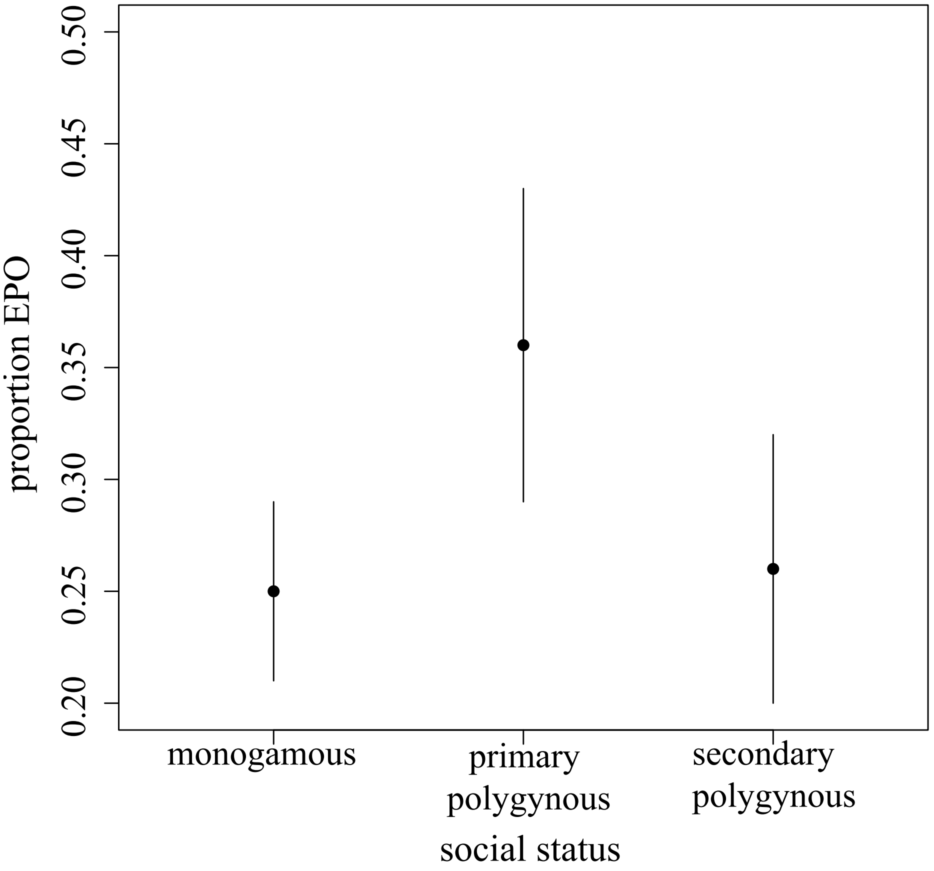

Figure S10. The relationship between the proportion of extra-pair offspring (P_{EPO}) in a brood and social status, defined as monogamous (one female per male territory); primary polygynous (two females per male territory but the first nest to hatch); secondary polygynous (two females per male territory but the second or third nest to hatch). Points indicate the mean across observed broods, and bars indicate the 95% CIs.

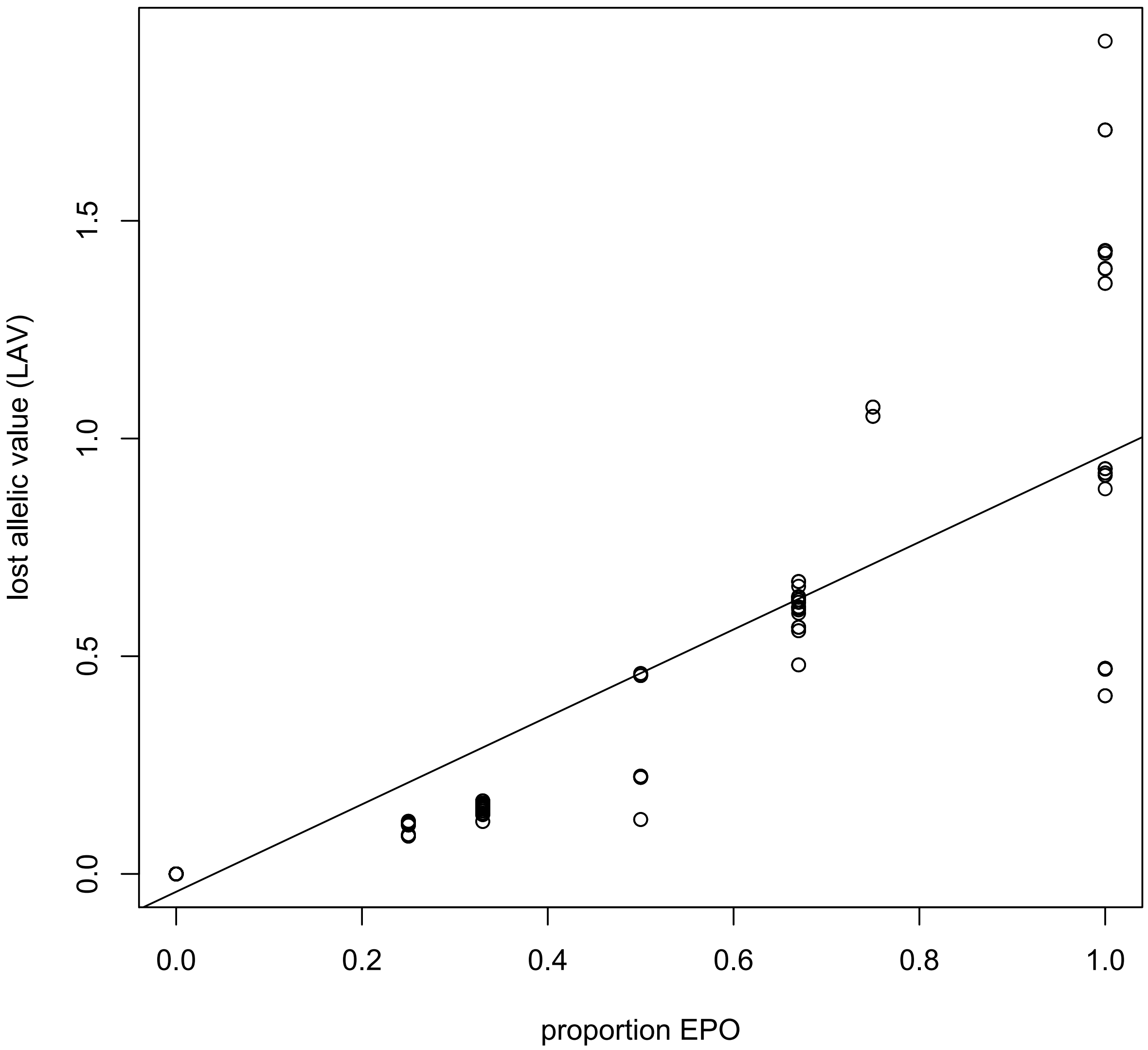

Figure S11. Relationship between lost allelic value (LAV) and the proportion of extra-pair offspring (P_{EPO}) in a brood. Points denote broods and the line denotes the linear regression.

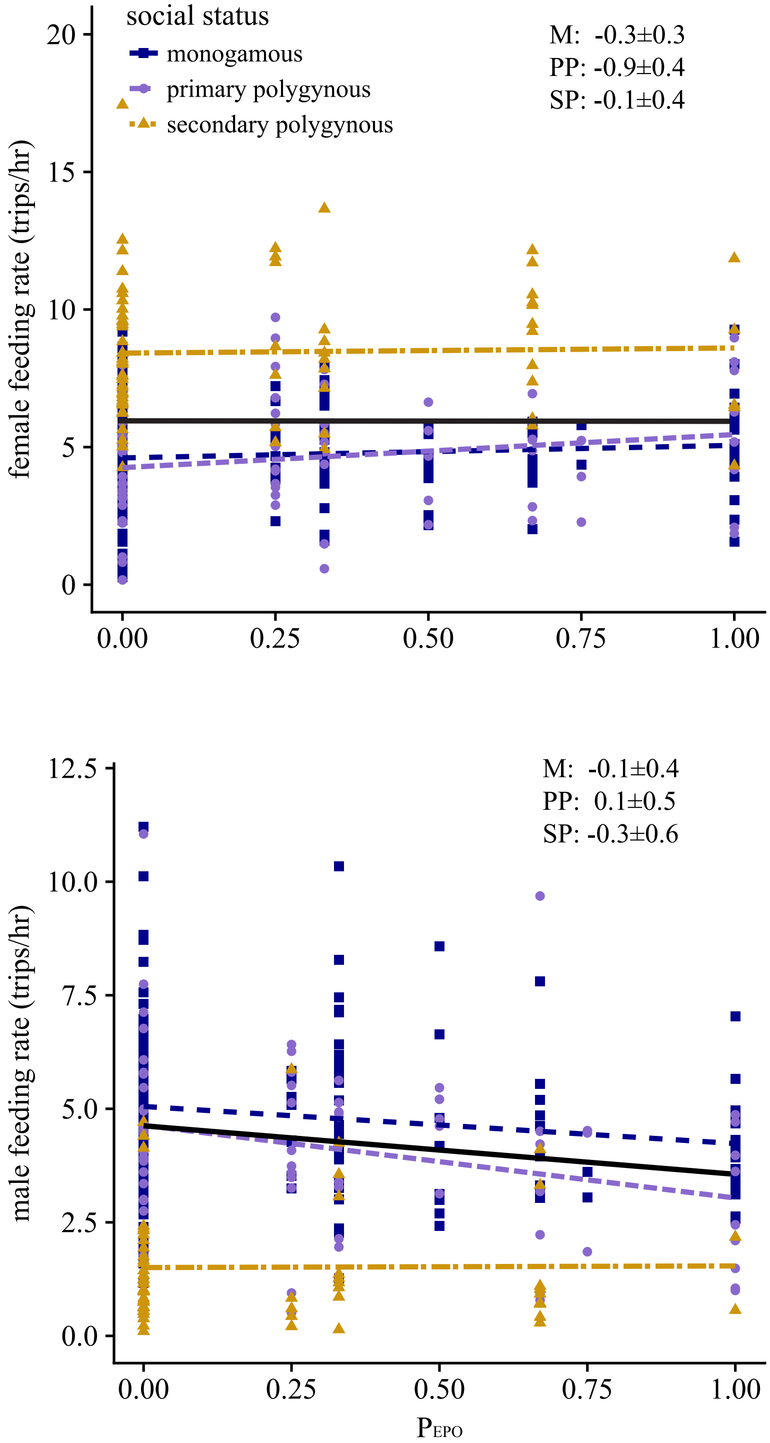

Figure S12. Relationships between (a) female and (b) male song sparrow parental feeding rates (trips/hr) and the proportion of EPO in the brood (P_{EPO}). Colours indicate nest social status (monogamous: blue; primary polygynous: purple; secondary polygynous: yellow). Points

represent observation sessions. Lines show predicted regression of feedings rate on P_{EPO} overall (black), and across each social status. Numbers are the estimates ± SE from the P_{EPO} by social status interaction model, where “M”, “PP” and “SP” are monogamous, primary and secondary polygynous nests, respectively. Y-axes are on different scales for males and females.

Appendix S6: Supplementary results

Table S1: Null model variables and results from the null model.

Table S2: Results from sets of models that contain additive or interactive effects of total allelic value (TAV), TAV standardized within brood size (TAV_z) and brood size (BS) on male and female feeding rates (trips/hr).

Table S3: Results from each models examining the effects of lost allelic value (LAV), the proportion of extra-pair offspring in a brood (P_{EPO}), kinship (k_{ij}), male and female coefficients of inbreeding (f_i and f_j) in comparison to the null model.

References

1. Nietlisbach P, Camenisch G, Bucher T, Slate J, Keller LF, Postma E. 2015 A microsatellite-based linkage map for song sparrows (*Melospiza melodia*). *Molecular Ecology Resources* **15**, 1486–1496. (doi:10.1111/1755-0998.12414)

2. Reid JM, Keller LF, Marr AB, Nietlisbach P, Sardell RJ, Arcese P. 2014 Pedigree error due to extra-pair reproduction substantially biases estimates of inbreeding depression. *Evolution* **68**, 802–815.

3. Reid JM, Arcese P, Bocedi G, Duthie AB, Wolak ME, Keller LF. 2015 Resolving the conundrum of inbreeding depression but no inbreeding avoidance: Estimating sex-specific selection on inbreeding by song sparrows (*Melospiza melodia*). *Evolution* **69**, 2846–2861.

4. Sardell RJ, Keller LF, Arcese P, Bucher T, Reid JM. 2010 Comprehensive paternity assignment: genotype, spatial location and social status in song sparrows, *Melospiza melodia*. *Molecular Ecology* **19**, 4352–4364. (doi:10.1111/j.1365-294X.2010.04805.x)

5. Reid JM, Duthie AB, Wolak ME, Arcese P. 2015 Demographic mechanisms of inbreeding adjustment through extra-pair reproduction. *Journal of Animal Ecology* **84**, 1029–1040. (doi:10.1111/1365-2656.12340)

6. Keller LF. 1998 Inbreeding and its fitness effects in an insular population of song sparrows (*Meospiza melodia*). *Evolution* **52**, 240–250.

7. R Core Development Team. 2018 *R: A language and environment for statistical computing. R Foundation for Statistial Computing*. Vienna, Austrai. See http://www.R-project.org.

8. Reid JM, Bocedi G, Nietlisbach P, Duthie AB, Wolak ME, Gow EA, Arcese P. 2016 Variation in parent-offspring kinship in socially monogamous systems with extra-pair reproduction and inbreeding. *Evolution* **70**, 1512–1529. (doi:10.1111/evo.12953)
